# Perceptions and experiences of pregnant women about routine HIV testing and counselling in Ghimbi town, Ethiopia: a qualitative study

**DOI:** 10.1186/s13104-017-2423-1

**Published:** 2017-02-16

**Authors:** Israel Mitiku, Adamu Addissie, Mitike Molla

**Affiliations:** 1 Department of Public Health, College of Medicine and Health Sciences, Wollo University, P. O. Box 1145, Dessie, Ethiopia; 2 School of Public Health, College of Health Sciences, Addis Ababa University, Addis Ababa, Ethiopia

**Keywords:** Routine, HIV, Opt-out, Pre-test counselling, Qualitative

## Abstract

**Background:**

Ethiopia has implemented routine HIV testing and counselling using a provider initiated HIV testing (‘opt-out’ approach) to achieve high coverage of testing and prevention of mother-to-child transmission of HIV. However, women’s perceptions and experiences with this approach have not been well studied. We conducted a qualitative study to explore pregnant women’s perceptions and experiences of routine HIV testing and counselling in Ghimbi town, Ethiopia, in May 2013. In-depth interviews were held with 28 women tested for HIV at antenatal clinics (ANC), as well as four health workers involved in routine HIV testing and counselling. Data were analyzed using the content analysis approach.

**Results:**

We found that most women perceived routine HIV testing and counselling beneficial for women as well as unborn babies. Some women perceived HIV testing as compulsory and a prerequisite to receive delivery care services. On the other hand, health workers reported that they try to emphasise the importance HIV testing during pre-test counselling in order to gain women’s acceptance. However, both health workers and ANC clients perceived that the pre-test counselling was limited.

**Conclusions:**

Routine HIV testing and counselling during pregnancy is well acceptable among pregnant women in the study setting. However, there is a sense of obligation as women felt the HIV testing is a pre-requisite for delivery services. This may be related to the limited pre-test counselling. There is a need to strengthen pre-test counselling to ensure that HIV testing is implemented in a way that ensures pregnant women’s autonomy and maximize opportunities for primary prevention of HIV.

**Electronic supplementary material:**

The online version of this article (doi:10.1186/s13104-017-2423-1) contains supplementary material, which is available to authorized users.

## Background

HIV testing practices have changed dramatically since the advent of large-scale antiretroviral treatment (ART) programs [1]. HIV testing and counselling (HTC) is a critical opportunity for pregnant women to receive HTC and hence prevention of mother to child transmission (PMTCT) of HIV [2]. Despite scaling-up of HCT services over the last decades, most HIV patients globally and particularly in resource poor countries are unaware of their status [3]. In an attempt to increase HIV test rates, the joint United Nations Programme on HIV/AIDS (UNAIDS) and World Health Organization (WHO) have recommended the implementation of routine HIV testing, also termed provider-initiated testing and counselling (PITC), in all health facilities of countries with generalized HIV epidemics [4].

The traditional approach has been client-initiated HCT, in the form of voluntary counselling and testing (VCT) [5, 6]. The ‘routine’ offer of HIV testing in health facilities, in contrast to VCT, is recommended by health care providers to persons attending health care facility as a standard component of medical care [4, 5]. Offering testing to all individuals seeking health care services is assumed to increase HIV test rates and thus increase access to treatment and prevention interventions including the PMTCT programme [7–9].

Ethiopia initiated the programme for PMTCT in 2001 using VCT approach [10]. Following the initiation of the UNAIDS and WHO recommendations, Ethiopia issued a guideline on routine counselling and testing for HIV using an opt-out approach, which was institutionalized since 2007 [11]. HIV testing and counselling became integrated into ANC, childbirth and postpartum services to achieve high coverage of HIV testing and PMTCT. Under this approach, all pregnant women seeking maternal care are tested except those who actively decline. According to the guidelines, women must receive adequate pre-test information on which to base a voluntary decision and the woman’s right to decline should be preserved [4, 11].

Routine HIV testing and counselling has been associated with increased testing rates among women attending ANC in many African settings [7, 9]. The proportion of mothers who accepted HIV testing in Ethiopia has increased from 57% in 2006 to 92% in 2010 [12]. However, the role of this testing model has been criticized from an ethical and human rights perspective for paving the way to neglect of informed consent [13, 14], and for reducing the amount of counselling that accompanies the HIV test [15]. Some studies have indicated that routine HIV testing is perceived as mandatory among pregnant women, and thus the opportunity to decline testing could be beyond their reach [16, 17]. The very establishment of opt-out HIV testing policy could also send a message that the test is institutionally sanctioned and being tested for HIV is the correct thing to do [13]. There is a concern that pregnant women exert their decision making power by not coming back for their test results which undermines the effectiveness of the whole package of PMTCT program [18, 19].

It is important to understand how routine antenatal HIV testing is perceived and experienced to ensure that this policy is implemented in a manner that retains trust and a minimum of the patients’ rights, and pre-test counselling is given to maximize access to HIV prevention. However, studies conducted so far in Ethiopia emphasized on the uptake of HIV testing, with relatively little attention to how routine HIV testing is perceived and experienced by pregnant women. Thus, the current study aimed to explore perceptions and experiences of routine HIV testing and counselling among pregnant women in Ghimbi town, Oromia region, Ethiopia.

## Methods

### Ethiopian national PMTCT guideline

Ethiopia has adopted the WHO/UNICEF/UNAIDS 4-pronged PMTCT strategy as a key entry point to HIV care for women, men and families in 2001. In 2007, Ethiopian government issued revised PMTCT guideline that promotes integrated and “Opt-Out” approaches as the most appropriate strategy for expanding national access and sustainability of PMTCT services in the country. Routine provider-initiated HIV counselling and testing using the opt-out approach is recommended for all clients seen within the context of maternal care. According to the guideline, clients are given pre-test information in a group or individually on HIV/AIDS and PMTCT and are told that their routine antenatal laboratory tests will include HIV test. The provider also must inform the client that she has the right to say “no” (to opt out), and this decision by no means affects the services she will get from the health facility.

Compared to other approaches, routine provider-initiated HIV counselling and testing using the opt-out approach for all pregnant women has resulted in greater acceptability, and increased opportunity to prevent MTCT [11]. As compared to the 2006 figures, the proportion of ANC clients provided with HIV counselling services at PMTCT sites and the number of HIV positive pregnant mothers identified and the proportion of have increased by more than threefold in 2010. The prevalence of HIV among those pregnant mothers who underwent HIV testing has decreased from 8% in 2006 to 2% in 2010 [12].

### Study setting

The study was conducted among pregnant women attending ANC clinics of one health center and one hospital in Ghimbi town. Ghimbi town is situated in West Wollega Zone of Oromia Regional State, 441 km west of Addis Ababa, the capital city of Ethiopia. Ghimbi town is the capital of Ghimbi district, which is one of the 21 districts in the Zone. Based on the 2007 National census the total population of the district was about 74,623, of which 30,981 were from Ghimbi town. Women of reproductive age (15–49 years) constitute about 28% of the total population in the town [20]. In 2013, there were two public health institutions, including one primary hospital and one health center, providing HIV testing and counselling for pregnant women and offering ART and other necessary care for HIV positive women and their infants in Ghimbi town and surrounding areas.

### Study design

A qualitative research design using in-depth interviews (IDIs) was employed to explore perceptions and experiences of pregnant women with routine HIV testing and counselling provided as part of ANC. IDIs was considered to be an appropriate method as the aim of the study was to elicit individual experiences and perceptions with HIV testing and counselling process. Key informant interviews were conducted with health workers who were involved in routine HIV testing and counselling to explore their views on PITC with particular emphasis on pertest counselling and consent process.

### Study population and sampling

The study population included women who were attending ANC for the first time during the current pregnancy and tested for HIV at the two public facilities in May 2013. We conducted a total of 28 IDIs until we reached information saturation where we felt that adding more interviews would not bring forth any new information. Women were selected purposively to consider the variety of participants in terms of parity, educational level and experience of routine antenatal HIV testing. They were interviewed after going through HIV counselling and testing but before receiving the test result. Four IDIs were conducted with ANC staffs who were involved in routine HIV testing and counselling. Three of the interviewees were midwives and one was a clinical nurse. Two health workers were recruited from public hospital and two from health center.

### Data collection

The interviews were conducted in Afaan Oromo (the local language) at a place that provided optimum privacy and tape recorded after consent was received. A pretested interview guide was used to explore women’s perceptions and experiences with routine HIV testing and counselling. The guide included questions related to knowledge and perceptions about routine HIV testing and counselling, and experiences with pre-test counselling and consent process. Probing questions were included in the interview guide in case the responses of the participants are superficial and/or the answers are conflicting (Additional file 1). Interviews took between 20 and 45 min to complete. The interviews were moderated by the first author and attended by one health professional who took notes, both fluent in the local language and familiar with qualitative research methods.

### Data analysis

Preliminary data analysis was concurrent with data collection and evolved throughout the data collection and analysis period. Debriefing was conducted at the end of each data collection day to share preliminary findings and identify areas to be explored more. Tape recordings were transcribed verbatim in local language and translated into English. Further data analysis was conducted by two members of the research team. The analysis involved multiple reading of transcripts to understand the data to identify emerging themes. The first and the last authors independently coded the transcribed data using an inductive approach and the codes were compared for consistency. Transcripts were coded line by line and data were analyzed through thematic content analysis using the qualitative research analysis software, Open Code 3.6.2.0. The codes were compared for similarities and differences, and themes were developed. Salient quotes were used to express the experiences and perceptions of women in the study findings.

## Results

### Characteristics of study participants

The age of the participants ranged between 15 and 35 years and the majority had completed primary education. Most of the women were multiparas and housewife. All of them were married and living with partner (Table 1).

**Table 1:** Socio-demographic characteristics of pregnant women (n = 28), Ghimbi town, West Ethiopia

Variable	Number
Age group	
15–19	4
20–24	13
25–29	8
30–35	3
Residence	
Rural	16
Urban	12
Education	
Illiterate	6
Primary education	15
Secondary and above	7
Number of previous pregnancies including the current	
One	10
More than one	18
Occupation	
Housewife	22
Merchant	1
Government employee	5

### Women’s perceptions and experiences with HIV testing

In this section, results of our analysis on women’s experiences and perceptions about routine HIV testing and counselling are presented using four themes emerged from the study: HIV testing during pregnancy is beneficial; inadequate pre-test counselling; HIV testing perceived as a compulsory for all women; and HIV testing perceived as a pre-requisite to other health services (Table 2).

**Table 2:** Thematic presentation of pregnant women’s perceptions and experiences of HIV testing as part of antenatal care at antenatal clinics in Ghimbi town, West Ethiopia

Themes	Sub-themes
HIV testing during pregnancy is beneficial	HIV status
	Protect unborn babies
Inadequate pre-test counselling	Client overload
	Post-test counselling important
	Prevention mother-to-child transmission of HIV is impossible
HIV testing is compulsory for all pregnant women	HIV testing for all pregnant women
	Government law
	Emphasis on the benefits of HIV testing
	Providers’ stance to protect unborn baby
HIV testing perceived as a prerequisite to other health services	Denial of health services
	Low quality of health services

### HIV testing during pregnancy is beneficial

Throughout the interviews, almost all women expressed the benefits of HIV testing during pregnancy. Two major benefits of routine HIV testing that were brought up continuously during the interviews were ‘knowing one’s status and protection of unborn babies from HIV infection’. Most women described that knowing a pregnant woman’s HIV status is essential to protect her unborn baby as there is access to treatment.

It is very good to get blood tested for HIV. It is good to know your status. A woman will take care of her child if she knows her HIV status… (18 years).

Participants reported that they knew the benefits of HTC during pregnancy from different sources including community education and mass media.

They have been teaching us coming to the kebele and I also heard from a television that if a pregnant woman gets tested for HIV, it is possible to prevent the transmission of the virus to the baby as the woman is seen by health workers and get treatment…. (34 years).

ANC visits for previous pregnancies were also mentioned as one of the sources of women’s awareness that HIV testing is performed at the heath institution and their knowledge on the benefits of HIV testing. As elaborated by one woman:

Whenever you come for follow up (ANC), they (health workers) first test your blood for HIV. In case I am infected with HIV, they have something to do (give treatment)… (27 years).

### Inadequate pre-test counselling

In the provider-initiated opt-out HIV testing guidelines, it is stated that, clients should receive pre-test information in a group or individually on HIV/AIDS and PMTCT. Our in-depth interviews among pregnant women found that the pre-test counselling accompanying the test was limited.

She (the midwife) drawn my blood to check my status; She told me nothing… (23 years)

They (health care providers) told us nothing. We gave blood based on what we have been hearing in the past. She came from outside and called my husband and said, "we need to test your blood for HIV", we accepted… (20 years).

This limited pre-test counselling was reflected by some women’s lack of knowledge about the possibility of protecting children born to HIV positive women as one woman described below:

If the mother is healthy, the baby will also be healthy. Pregnant woman’s knowledge of her HIV status has no benefit to the baby if she has already been infected with the virus… (29 years).

Though, health workers acknowledged the importance of providing pre-test information they reported that they provide limited pre-test counselling due to large number of clients.

Counselling is important. If there are few clients we explain to them some introduction about PMTCT and then do HIV testing. Of course, counselling is missed. If we take longer time in providing pre-test counselling, others who wait outside will complain… (Health worker).

In addition, another health worker emphasized on the importance of post-test counselling for HIV positive women compared to for those who are HIV negative women. She believed that there is no need to counsel HIV negative women as the goal of HIV testing is to prevent mother to child transmission of HIV.

The goal that needs to be emphasized is to save the baby. If the woman becomes HIV positive, there will be more counselling after the test. (Health worker).

### HIV testing was perceived as compulsory for all pregnant women

According to the current guidelines, women must be explicitly informed of their right to refuse testing. In this study, HIV testing was not however perceived as a choice, but rather as a compulsory service for all pregnant women. Some participants stated that pregnant women are tested for HIV as part of ANC, along with other routine examining procedures.

Blood is taken from this (showing her finger) for such things (HIV testing). HIV testing is performed every year when we are pregnant and come for follow-up… (26 years).

Another woman elaborated the perception that routine HIV testing is a government policy;

They (health workers) simply told me to test my blood for HIV. I didn’t ask why they needed my blood since I thought that the test is a must… (32 years).

When asked if they know that routine HIV testing provided as a part of ANC is a choice, only few knew that HIV testing is optional. Those who knew it was optional indicated their views as follows:

It is based on my will. It is known that HIV testing has a benefit, but it is possible to refuse. All women should be tested but should be on their will. I should be tested for HIV based on my interest… (25 years).

Health workers also approved the compulsory nature of the HIV testing as follows:

It is well documented that HIV testing should be voluntary, as stated in the guidelines. But you (health worker) emphasize on the importance of HIV testing when you give counselling, and you must push them to accept… (Health worker).

### HIV testing perceived as a pre-requisite to other health services

We asked our participants if refusal of HIV testing impacts the services that a woman gets from the health facility. Some women were concerned about their inability to decline the HIV test. They thought that refusal of HIV testing could result in a denial of other health services including delivery service:

I can’t refuse. Refusing HIV testing will create a problem to the woman. If she refuses what they (health care providers) ask her to do, they may not help her when she come back for delivery… (27 years).

Our participants explained that a woman who refused will not be equally treated during delivery with those women who accepted HIV testing. In view of this, one of the women explained:

Refusal of HIV testing has a problem during delivery. They will not equally serve two women, one who refused and the other who accepted the offer of HIV testing. They will not be equally served. She would be in trouble if she faces difficulty during delivery since she refused the test… (30 years).

## Discussion

This study found out that routine HIV testing and counselling is acceptable to pregnant women. Most women had awareness of routine antenatal HIV testing prior to coming for ANC visit. Women mentioned that they learned about HIV testing and counselling and its benefits from different community education, mass media, as well as during their previous antenatal visits for the last pregnancy.

In this study, women valued routine HIV testing and counselling as it provides them an opportunity to learn their status and protect their unborn baby since those found to be HIV positive could access treatment. Thus, acceptability of HIV testing in the study setting could in part be an indication of women’s strong desire to protect their unborn child as well as concern for their own health. Other studies in Africa have also identified women’s need to protect their children and the concern for own health as key reasons for acceptance of HIV testing during pregnancy [9, 21, 22]. Nevertheless, women who were aware of the voluntary nature of opt-out HIV testing were against mandatory HIV testing. The assertion that women should have the right to refuse HIV testing has been documented in other African settings [23]. This finding is line with global guidelines and Ethiopian HIV testing policy that clients’ right to informed consent should be respected [11].

In routine provider-initiated HIV testing and counselling, as stipulated in global and national guidelines, clients must receive pre-test information in a group or individually on HIV/AIDS and PMTCT [4, 11]. In our study, however, the majority of pregnant women, along with the data from the interviews with health workers portray that pre-test counselling was limited. Similarly, in a study done in Addis Ababa, providers were observed taking blood samples for HIV test from clients without pre-test counselling [24]. Health care providers mentioned that counselling is particularly important for HIV positive pregnant women, implying that counselling is not as such critical if a woman is HIV negative. This finding suggests that counselling is not prioritized for the majority of people who test negative, which indicates missed opportunities for primary prevention of HIV. Pre-test counselling is vital for pregnant women to ensure that they understand the implications of negative or positive test results for themselves, their partners and their unborn children [25, 26].

In this study, client load was raised as a barrier to pre-test counselling. In the ANC environments, health care providers were overwhelmed by the high numbers of women attending come to their clinics for testing. This was observed to have affected the quality of counselling services hampering the delivery of the full package of PMTCT services. Similarly, studies in sub-Saharan Africa countries had demonstrated limited pre-test counselling related to the small number of health workers in facilities [21, 27]. To this effect, strategies including increasing the number of health workers and using lay counsellers that may help to ensure that provider-initiated HIV testing and counselling take place in the manner it was intended to [28, 29].

The Ethiopia PMTCT guidelines recommend that providers must explicitly inform the client that she has the right to say “no” (to opt-out), and that this decision by no means affects the services she will get from the health facility [11, 30]. In this study, however, some women do not perceive HIV testing as a choice, but rather as a compulsory service for all pregnant women. The perception that HIV testing provided as part of ANC was compulsory, has also been documented in other African settings [16, 21]. Some women perceived this approach as a government policy intended to protect children from HIV infection. For such women, the implication here is that acceptance of HIV testing could be considered as a compliance with what they perceived as a government law. Health workers’ emphasis on the benefits of HIV testing during pre-test counselling we found in this study, could have downplayed women’s perceptions of the possibility of not testing, which is in congruent with a study from Uganda [21]. Similarly, a study from four African countries revealed some providers underlying their stance as the moral imperative to protect the unborn child [23]. Moreover, some women in our study perceived HIV testing as a prerequisite to receive other health services including delivery services. Similar findings have been documented in other African settings [17, 23]. This is a fundamental shortcoming of unclear pre-test counselling as entirely eliminating pre-test counselling or providing insufficient information limit the opportunities for ensuring informed consent [26, 30].

There are reports that women express their autonomy by not coming back for their test results. The implications for PMTCT could be that pregnant women accept to be HIV tested but fail to return for the test results as they realize that they are unprepared for the consequences as it has been reported that pregnant women would express their autonomy by not coming back for their test results [18, 19, 31].

The use of qualitative methods using IDIs enabled us to have detailed understanding regarding women’s perceptions and experiences of the provider initiated opt-out HIV testing provided as part of ANC. The IDIs with health care providers enabled us to triangulate the findings from interviews with pregnant women which could indicate the trustworthiness of the findings. Our study had some potential limitations. The first limitation of the study is that experiences of pregnant women who declined HIV testing were not gathered as none of the antenatal attendees refused the test. Facility environment could influence respondents’ responses about health care services. We do not however think that social desirability bias has much effect on the findings, based on the fact that data collectors were independent of health care providers of the health facilities included in the study.

## Conclusions

In conclusion, routine HIV testing and counselling provided as part of ANC is well acceptable among pregnant women in the study setting. However, there is a sense of obligation as women felt the HIV testing is a pre-requisite for delivery services. Our findings suggest that the voluntary nature of routine HIV testing is not fully understood. This may be related to the limited pre-test counselling. There is a need to strengthen pre-test counselling to ensure that HIV testing is implemented in a way that ensures pregnant women’s autonomy and maximize opportunities for primary prevention of HIV.

## Additional file

**Additional file 1.** Interview guide.
