# Supplementary material for: Perceptions and experiences of pregnant women about routine HIV testing and counselling in Ghimbi town, Ethiopia: a qualitative study
Source: BMC Res Notes. 2017 Feb 16;10:101. doi: 10.1186/s13104-017-2423-1 (PMC5314483; doi:10.1186/s13104-017-2423-1)
Supplement: Supplementary file 1 — Additional file 1. Interview guide. [file 13104_2017_2423_MOESM1_ESM.docx]

## ****Additional file 1****

**Interview guide.docx**

1. Is it important for a pregnant woman to be tested for HIV? If yes, probe for benefits of HIV testing to the mother, baby, husband and the family in general.
2. Do you think that mother to child HIV transmission could be prevented? If yes, how?
3. Have you been told the reasons for taking blood when you are expecting a baby? Do you understand what the health workers want to look at in the blood? (Probe for woman’s understanding of what health workers test blood for: Any infections she may pass on to the baby—HIV
4. Before coming to the clinic, did you think that you might be offered a blood test for HIV? If no, was it a surprise to be asked? If yes, did you know before? How did you find out? Probe: Had you made up your mind whether you would accept or not?
5. Did the midwife ask you if you would agree to have your blood tested for HIV infection? Probe: What did she say?
6. Did she explain to you why they wanted to test your blood now, when you are expecting a baby? Probe: What did she say?
7. What did you think about being asked to agree to a blood test for HIV infection? Probe: Did you think it was a good idea? If yes, why do you say that?
8. Did you agree to have a blood test for HIV? If, did you feel that you could say no? If not, Probe: Why not? If no, did you feel that it was hard to refuse? If yes, Probe: why? what would happen if a woman did not want to be tested for HIV?
9. What made you decide whether or not to have the blood test? Were you satisfied that you had adequate information to make a decision about HIV testing?
10. How do you feel about the decision you have made?
